# Developing synthetic biology for industrial biotechnology applications

**DOI:** 10.1042/BST20190349

**Published:** 2020-02-20

**Authors:** Lionel Clarke, Richard Kitney

**Affiliations:** 1 UK Synthetic Biology Leadership Council, London, U.K.; 2 Department of BioEngineering, Imperial College London, London, U.K.; 3 School of Chemistry, University of Manchester, Manchester, U.K.; 4 BionerG, Chester, U.K.; 5 EPSRC National Centre for Synthetic Biology and Innovation, (‘SynbiCITE’), London, U.K.; 6 Institute of Systems and Synthetic Biology, Imperial College, London, U.K.

**Keywords:** bioeconomy, industrial biotechnology, synthetic biology

## Abstract

Since the beginning of the 21st Century, synthetic biology has established itself as an effective technological approach to design and engineer biological systems. Whilst research and investment continues to develop the understanding, control and engineering infrastructural platforms necessary to tackle ever more challenging systems — and to increase the precision, robustness, speed and affordability of existing solutions — hundreds of start-up companies, predominantly in the US and UK, are already translating learnings and potential applications into commercially viable tools, services and products. Start-ups and SMEs have been the predominant channel for synthetic biology commercialisation to date, facilitating rapid response to changing societal interests and market pull arising from increasing awareness of health and global sustainability issues. Private investment in start-ups across the US and UK is increasing rapidly and now totals over $12bn. Health-related biotechnology applications have dominated the commercialisation of products to date, but significant opportunities for the production of bio-derived materials and chemicals, including consumer products, are now being developed. Synthetic biology start-ups developing tools and services account for between 10% (in the UK) and ∼25% (in the US) of private investment activity. Around 20% of synthetic biology start-ups address industrial biotechnology targets, but currently, only attract ∼11% private investment. Adopting a more networked approach — linking specialists, infrastructure and ongoing research to de-risk the economic challenges of scale-up and supported by an effective long-term funding strategy — is set to transform the impact of synthetic biology and industrial biotechnology in the bioeconomy.

## Introduction

Synthetic biology and industrial biotechnology are recognised as key platforms for the growth of the Bioeconomy, yet synthetic biology is a relatively recent development and remains unfamiliar to many outside the field. Recognising the relationship between these two distinct yet complementary technologies is important, to support their ongoing development and to ensure that the strategic benefits of a technology-driven bioeconomy can be delivered.

Industrial biotechnology (IB), defined as ‘using enzymes and microorganisms to make bio-based products in sectors such as chemicals, food and feed, detergents, paper and pulp, textiles and bioenergy (such as biofuels)’ [1] generally involves working with these natural systems to maximise and optimise existing biochemical pathways that are used in manufacturing. Modern IB emerged as a field in the 1970s. Detergent enzymes were an early commercial example, and still account for 30% of an industrial enzyme market worth ∼$5bn annually [2].

Synthetic biology originated around the turn of the 21st century, inspired in part by considering analogies with the significant advances previously achieved in electronic microcircuit design, and facilitated by the rapid decrease in the costs and time to collect DNA sequencing data following the completion of the Human Genome Project (HGP). It was effectively established to address ‘what it would take to advance the deliberate and rational engineering of living systems’ [3]. At the heart of synthetic biology is BioDesign, applying the engineering principles of modularity, standardisation and characterisation/abstraction to improve the practical capacity to programme and construct biological systems to produce specific human-designed outputs with predictable properties and functions.

The commercial translation of synthetic biology is advancing very rapidly, primarily via investments in start-up companies and SMEs providing tools, services and products to market. The ‘Synthetic Biology UK — A Decade of Rapid Progress’ brochure, published online in July 2019 [4], provides an illustrative set of UK-based examples.

## The bioeconomy driver

Heightening awareness of significant global challenges — including the need to actively mitigate climate change, to improve food security, to reduce ocean pollution, to conserve biodiversity and to establish more sustainable manufacturing supply chains — combined with increasing societal concerns over the provenance of food and natural product supply chains, is stimulating significant growth in investments into the development of technological options. To limit future global temperature increases to no more than 1.5°C will require a significant and rapid reduction in the currently world-wide dependence on fossil-based feedstocks for fuel and chemicals [5] necessitating radical changes to established industrial operations.

The overall bio economic driver is towards a sustainable, low carbon bio-based economy. This represents a new model for industry and the economy that uses renewable biological resources sustainably to produce food, energy and industrial goods. The sustainable production of molecules from biomass feedstocks as the component building blocks of future medicines, chemicals, materials and liquid fuels — exploiting the untapped potential stored within millions of tons of biological waste and residual materials — is core to the future circular economy. The UK Bioeconomy Strategy, launched in December 2018 [6], provides a plan to help address such challenges. In it, IB and synthetic biology are recognised as providing a unique platform for growth and the creation of a more sustainable future for all. Mechanisms to assist the acceleration of growth via synthetic biology and IB — broadly defined as ‘Engineering Biology’ — have been recently outlined in a report published by the UK's Royal Academy of Engineering [7]

## Developing the synthetic biology platform and applications

Consortia and partnerships play an important role. Following recommendations in the 2012 UK Roadmap [8] and further developed in the 2016 Biodesign strategy [9], six new synthetic biology research centre (SBRCs) hubs and additional infrastructures were established [10] to complement the original CSynBI research centre and to establish SynbiCITE, the national translation centre [11], plus a Centre for Doctoral Training (a collaboration between the universities of Oxford, Warwick and Bristol) and four Biofoundries. Partnerships, linking not only these particular hubs but also numerous other research and development centres, have helped establish effective UK-wide networks of significant expertise. In the US in 2006 the NSF — under a 10-year grant — established a consortium of major universities and companies, Synberc with a primary goal to ‘develop the foundational understanding and technologies to build biological components and assemble them into integrated systems to accomplish many particular tasks’. This has now been replaced by the engineering biology research consortium (EBRC) [12]. Recently, a new Centre for Doctoral Training in BioDesign Engineering [13] (a collaboration between Imperial College, the University of Manchester and University College London), and a national Future Biomanufacturing Research Hub (FBRH) Centre [14] have been established in the UK, building upon the infrastructural investments into synthetic biology made to date.

Synthetic biology facilitates the development of applications not only in IB, but also in health biotechnology, agriculture, marine biotechnology and environmental. The underlying technology platform is essentially application-agnostic, but adheres to the engineering principles, the assimilation of automation and information technologies — maintaining a clear focus on the issues of precision, repeatability and robustness that must be addressed to facilitate the industrialisation of applications.

Standardisation is key. Guidelines for the use of standards relating to synthetic biological systems (PAS 246) were published in 2015 by the British Standards Institution (BSI) [15]. Ongoing developments include the CAD standard DICOM-SB, which facilitates the characterisation of biological components (e.g.‘Bioparts’) by the comprehensive capture of the data, metadata and notes associated with a characterisation experiment [16]. In 2018, Imperial College and the UK National Physical Laboratory established a Joint Centre of Excellence in Engineering Biology, Metrology and Standards to further engage with industry to help transform high-value manufacturing into high-value products [17]. Significant progress has been achieved in establishing internationally agreed technical standards. SBOL (synthetic biology open language) was adopted by ACS Synthetic Biology as a basis in 2016 [18]. Ongoing activities to establish standards at an international level include engagement with BioRoboost — a European H2020 Coordination Support Action project also including partners from USA, China, Japan and Singapore [19].

The engineering concept of assembling a fully functioning system from standard parts cannot be expected to be achieved by applying a simple ‘plug and play’ approach, due to the many interrelationships that occur within a living system [20]. A further engineering construct — the design-build-test-learn (DBTL) cycle — constitutes another fundamental feature of the process to discover and optimise the target system [21].

The establishment of increasingly high-throughput ‘Biofoundries’ (‘gene foundries’) to perform the relevant DBTL cycles not only speeds up (and reduces the cost of) identifying and optimising solutions, but generates opportunities for the application of machine learning and AI to further assist the process and to explore a much greater range of design space than would otherwise be feasible. The progression from wet laboratory activities to Biofoundries and the increasing use of BioCAD design will be a natural outcome of synthetic biology development. Recognising the importance of common standards to interoperability and the value of shared learning experience, SynbiCITE was a prime mover in the establishment of a Global Biofoundries Alliance (GBA), launched in May 2019. The initial cohort comprised the three prime movers (Imperial, Berkeley and NUS Singapore), plus the four other UK foundries and 15 other foundries from Europe, the US, Asia and Australia [22].

Whilst sequencing has become increasingly ubiquitous, due to massive reductions in costs and timescales since the HGP, other ‘pinch-points’ in the overall process such as the speed, sensitivity and costs of analysis and gene synthesis continue to be addressed. Examples include the ability to sort and analyse individual cells [23], faster and more robust gene-editing tools [24], and the generation of faster and more reliable synthesis techniques [25].

Significant efforts — for example, to develop greater understanding and control of intracellular and intercellular processes [26,27], the inter-cell portability of effective modularised functions [28] and the behaviour of microbial communities [29] — continue to inform the biodesign process. Scientists at the UK Centre for Mammalian Synthetic Biology in Edinburgh [30] have built a ‘tool box’ for cell biologists facilitating a wide range of biomedical and industrial applications, and could help reduce the need for animal experimentation. Open Plant, another UK SBRC, is pioneering the development of open tools and innovation in agritech research, IB and bioengineering services [31]. The ability to re-programme different hosts (‘chassis’) commonly used in industrial fermentations, such as yeast *Saccharomyces cerevisiae*, will be important. Significant progress towards designing and building the 16 synthetic chromosomes of yeast is already being achieved [32]. Recently, scientists at SYNBIOCHEM, the University of Manchester-based SBRC, have successfully demonstrated the effectiveness of the DBLT engineering biology approach — automating the biomanufacturing compound agnostic pipeline for the on-demand production of a diverse range of industrially relevant chemical building blocks [33].

## Translation, adoption, commercialisation

Synthetic Biology is more than just a research activity. It embraces the entire innovation pipeline from transformative technology platform to new applications and to the improvement of existing industrial products and processes.

Progress in Synthetic Biology may be described as ‘revolutionary’ in terms defined by Thomas Kuhn — by virtue of the transformative capabilities of the emergent toolkit and the rapid rate of increase in the number of practitioners becoming versed in the technology [22]. This is a key opportunity to develop innovative solutions, but also presents a potential stumbling block to its uptake by the wider IB community unfamiliar with the field. For example, chemical industries that currently operate using thermo-chemical processes of fossil-based feedstocks are now having to consider the inclusion of fermentation processes to use bio-based feedstocks as a more sustainable alternative in response to shifting customer interests [34].

Critical to commercial translation are the development of a skilled workforce and the effective derisking of target scale operations to assist potential industrial partners and investors alike. Spin-outs and start-ups currently appear to be the main channel of industrial translation [35]. Uptake by established corporates is more difficult to assess as there is no direct measure of internal R&D spends specific to synthetic biology. The rate and nature of industrial translation is also influenced by national cultural and operational differences. Over 80% synthetic biology academic research publications as identified by the Web of Science [36] in 2019 were generated by authors from just four countries: USA (36%), China (18%), UK (15%) and Germany (12%). As noted below, the relatively substantial research output from Germany is not reflected in start-up company private investments and implies different routes to industrial translation may also exist within Europe.

Companies offering synthetic biology-based tools and services support other SMEs and existing industries alike, operating either as basic service providers, or via more integrated partnership arrangements. New companies developing particular products may grow unilaterally or via mergers or acquisitions. The appropriate scale at which a new company needs to demonstrate its technology is highly dependent on the application. For high-value low volume products in healthcare, demonstration and accompanying IP at laboratory scale may be sufficient, whilst for high-volume, low margin chemical components, it may be necessary to demonstrate operations at an appropriately large scale or at least demonstrate confidence in its scaleability [37].

Because the US is world-leading in the establishment and financing of start-ups from its research base, monitoring its development in recent years provides a very useful indicator of the development of the field. SynbioBeta [38], based in San Francisco, has been tracking synthetic biology start-ups (predominantly US and UK based) and their associated funding over the past decade, whilst SynbiCITE [39] has been tracking UK synthetic biology start-ups for a similar period. Latest estimates are that the synthetic biology industry has attracted investments in excess of $12bn [40] in the past 10 years, predominantly within the US and UK.

**Figure 1.**
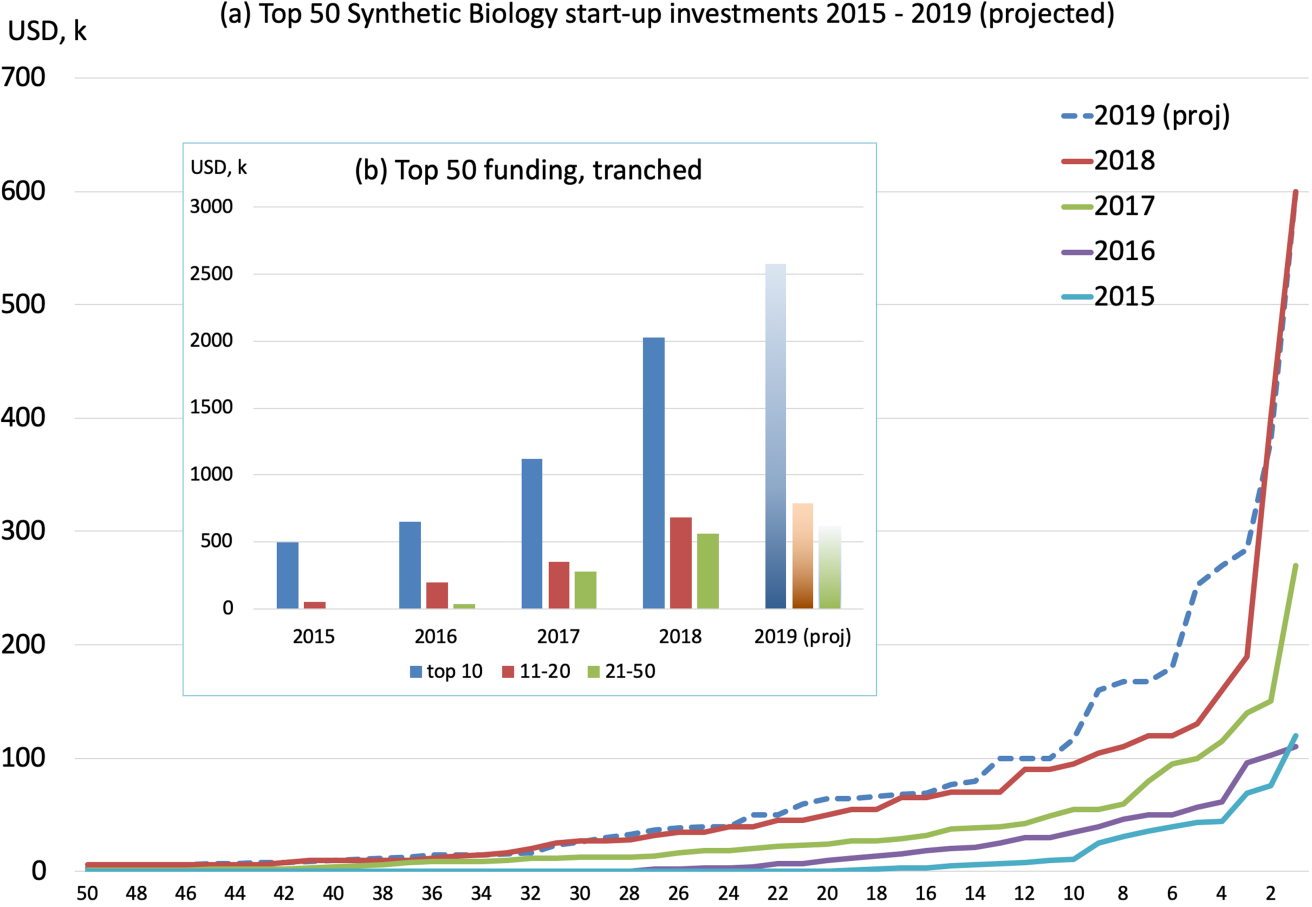
(**a**) top 50 start-up companies funded in each year 2015–mid-2019 (projected to year-end by doubling mid-year data) and (**b**) growth three tranches — top 10, next, 20, remaining 30 companies. Analysis drawn from SynBioBETA online data.

SynbioBeta online data records that private funding attracted annually by start-ups ranged between $175k and $610k in the period 2009–2015, but has grown rapidly since — reaching $3.8bn in 2018 — and currently on track to attract a similar level of funding in 2019. The year-on-year increase in total funding since 2015 reflects companies growing and maturing through successive funding rounds, whilst the flow of new start-ups continues to provide multiple options for future growth — Figure 1a. From 2015 to mid-2019, 178 individual start-up companies are identified as having received one or more rounds of funding exceeding $1 m.

Tracking the top 50 highest funded companies in each year, and projecting the mid-2019 data to a full year estimate, shows the steep increase in the size of funding rounds, IPOs and exits dominated by the top 10 recipients (note — these are not always the same 10 companies each year). The next tranche of 10 companies attract similar funding investments to the remaining thirty companies — Figure 1b.

The 2016 SynbiCITE start-up survey identified 146 UK-based synthetic biology start-ups, together raising over £564 m ($705 m) of private investment, with the number doubling every 5 years on average [41]. A 2019 update, focusing on the top 70 UK synthetic biology start-up companies indicates private investments totalling over $1bn. The SynbioBeta data records 25 UK-based companies receiving private funding $708 m in the period 2015 — mid-2019, and a further nine start-ups (receiving $261 m) in the remainder of the EU (predominantly in Switzerland, France and Denmark). The private investment growth trend is very consistent, despite the different origins of the SynbioBETA and SynbiCITE databases.

**Figure 2.**
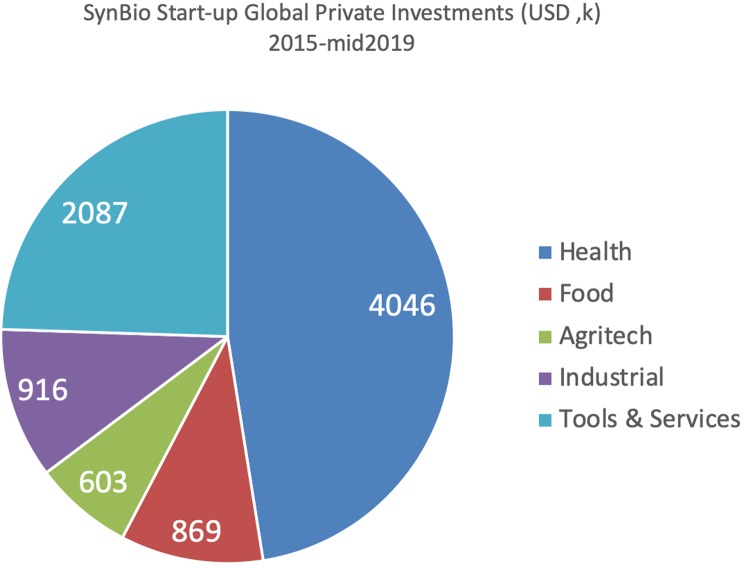
Synthetic iology start-up investments, segmented (from SynBioBeta online data).

Segmenting the 176 US/UK start-up companies (figure 2) shows that almost half (48%) of the investment is in health, followed by tools and services (24%), then industrial (11%), food (10%) and agritech (7%). Separately segmenting the synthesis of food (for human consumption as well as animal and fish food) from more conventional field-based agritech applications highlights the recent remarkably rapid and substantial increase in demand for alternative food options. This reflects a striking recent shift in consumer attitudes towards the role of genomics in aiding agriculture directly in the production of food whilst reducing pressure on the environment including land use and climate change, alongside improving animal welfare [36]. Antipathy towards GMOs has more than halved across the EU since 2011 [42]. Fifteen start-ups are currently developing alternative food forms, Impossible Foods [43] alone attracting $420m in 2019, as it continues to provide products such as plant-based burgers to commercial outlets in response to mounting consumer interest [44].

To date, UK start-ups have been more heavily focused on health (77%), then industrial (12%) and tools (10%), with limited investments to date in agritech or food (1%).

## Health biotechnology applications

Interest in developing health and healthcare applications, including pharmaceuticals, biologics and diagnostics, has provided a strong ‘market pull’ for the ongoing development of synthetic biology, especially in the UK [4]. From 2013 to2017, one-third of all biomedical start-ups in Europe were in the UK [45].

## Industrial biotechnology applications

In 2018, SYNBIOCHEM published a roadmap for the development of advanced materials via synthetic biology [46]. Compared with healthcare, shifting chemical and materials production from fossil-based to bio-based represents a far more significant challenge, both in developing the expertise in an otherwise unfamiliar technology, and in terms of the capital investment and long lead times generally required to meet large volume production targets. However, many chemical companies are recognising shifting customer requirements for low carbon alternatives [47], and encountering policy drivers, such as the commitment to establish a Circular Economy, which are shifting the economic balance of commercial options in favour of those that can convert and add value to waste streams. In 2011, the OECD identified the significant potential of IB to help address climate change, noting that synthetic biology may be ‘the vehicle that moves biotechnology into the economic mainstream’, but that it lacked adequate funding and required clear policy support [48]. This may be changing. Carlson estimates that 25% of all chemicals produced in the US are now biologically based [49].

The transition from fossil-based to bio-based feedstocks requires a very different approach to feedstock supply. Fermentation and biomass lend themselves to the development of smaller-scale, distributed operations, more akin to farm-based anaerobic digestion than large-scale petrochemical facilities. In 2018, Cambridge Consultants concluded that ‘the industry needs to shift from a ‘one company does it all’ model to a network of specialised companies, contributing to an integrated, efficient and competitive supply chain. Not only does this approach build expertise and allow companies to focus on their strengths, but it also reduces development risk and thereby builds investor confidence’ [50]. In June 2019 the EBRC, drawing upon inputs from ∼90 authors, published their technical research roadmap [51] to ‘enable next-generation production through sustainable, cost-competitive, flexible, and efficient manufacturing processes’ and to achieve the ‘scalable production of novel and existing products that are more sustainable and economically- and environmentally-friendly’.

Partnering and forming joint ventures have been commonly used to assemble the necessary resources and expertise and to help de-risk the technology prior to making major capital investments. Significant examples from the past include the partnership between DuPont and Genencor in 1997 to apply metabolic engineering to convert glucose to 1,3-propanediol (PDO), finally reaching commercial scale in 2006. In 2013, BASF partnered with Genomatica to produce bio-based 1,4-butanediol (‘bio-BDO’), yet to be commercialised at world-scale [52]. Genomatica reported a development period of 5–8 years to develop their single-step fermentation process prior to their partnership with BASF [53].

The ongoing development of synthetic biology for chemical processes is helping to accelerate (and reduce the costs of) such co-developments. Companies that have already developed commercial biotechnology processes may work directly with service providers such as Ingenza [54] to access latest technological techniques to achieve further optimisation, or to optimise or access improved enzymes, as provided by Oxford Biotrans [55]. Amyris has pioneered the entire development pipeline of a range of commercial applications, built upon its farnasene platform [56], whilst other spin-outs are now also beginning to establish novel platforms, such as C3 Biotech, a SYNBIOCHEM spin-out, which is commercialising new routes to biofuels, including the world's first fermentation route to bio-propane [57].

## Shifting consumer concerns, market pull

By virtue of its anticipatory approach, the development of synthetic biology has always been accompanied by the proactive consideration of its potential future impact and societal issues and responsibilities arising. Ultimately, the benefits of synthetic biology will not be delivered by technological advances alone, but by the generation of a comprehensive eco-system that address the breadth of societal, regulatory and economic issues — embracing both opportunities and risks — associated with its commercialisation.

Social scientists were engaged at the beginning in the earliest research programmes in the UK as summarised in the Royal Academy of Engineering Report published in 2009 [58]. A Public Dialogue was commissioned, which identified many important viewpoints [59]. A series of workshops spanning a wide range of considerations were convened by the scientific and engineering academies from the US, China and UK [60]. Key recommendations from the 2011 Public Dialogue, were adopted in the 2012 UK Synthetic Biology Roadmap, from which amongst other things the concept of responsible research and innovation (RRI) was formally embedded in the establishment of all subsequent Synbio Research Centres [9]. RRI is proactively embedded in UK research programmes, generating a collective awareness and being continually refreshed as new insights are gained and learnings made. To assist the adoption of these approaches more consistently and across the wider industrial community, a set of standards and guidelines are being developed in the form of a framework for Proportionate and Adaptive Governance of Innovative Technologies (PAGIT) [61], from which the BSI is currently developing a ‘Standard for Responsible Innovation’.

A hallmark of this predominantly market-sensitive culture, and a consequence of being largely driven by start-ups is the capacity to recognise and respond rapidly to shifting societal needs and concerns. It is evident from recent investments analysis that an increasing proportion of synthetic biology start-ups are not simply aiming to provide lower cost or modified versions of established products, but are focussing on more intractable issues, such as the removal of persistent micropollutants by Puraffinity (formerly CustoMem) [62], the development and supply of innovative biodegradable natural polymers to help reduce plastics polluting the environment by Biome Technologies [63], the use of engineered microorganisms engineered by Colorifix to fix dyes to textiles [64] with a less toxic process that reduces water use by 90%, and dyes released by 99%. Bolt Threads produces ‘spider-silk’ yarns [65]. Whilst delivering technological targets, this approach also appeals to elements in the fashion industry concerned about silk-worm welfare. Synthetic biology applications now address a wide range of the UN Sustainability Goals [66].

## Building an expert workforce

Synthetic biology may still be an unfamiliar discipline to many in IB and in the chemical, agritech and other sectors, but increasing numbers of trained students are entering the market, helping provide the expertise required to help identify and develop emerging options. Training programmes divide into two categories; namely university-based education and training and business orientated courses. Over 1000 postgraduates have been trained in synthetic biology in the UK since 2014 [4]. There is now quite a wide range of university-based courses at both the undergraduate and postgraduate levels that cover synthetic biology techniques using the bio design approach (i.e. design, build, test and learn or DBTL). These comprise wet and dry laboratory methods. In the case of dry laboratory: programming, life-size toolkits, libraries and database technologies. Wet laboratory techniques include: DNA assembly methods, gene editing and design of experiments. SynbiCITE, the National Translation Centre for Synbio in the UK, provides a range of business education and training opportunities including the 4-day MBA (‘More Business Acumen’) for synthetic biology entrepreneurs, and a customer facing course called Lean Launchpad — which addresses the issue of product development in relation to the customer base. Courses are delivered at both at its White City campus and in other regional centres [31]. Start-ups themselves are also playing a significant skills development role by training employees on the job. It is estimated that the roughly 150 UK start-ups to date now employ over 2000 staff [4]. Around 40 000 students worldwide have at least some practical experience of synthetic biology and its application through participation in iGEM [67].

Established equipment manufacturers and service suppliers are also fully engaged for example in the development of infrastructure such as equipping the Biofoundries. As synthetic biology contributes to and transforms manufacturing and services throughout the BioEconomy, so the benefits to an ever-wider range of jobs are being generated.

As noted, numerous applications are now being commercialised or becoming commercialisable, but it is also clear that a significant need for ‘a long tail’ of public funding is required, to continue generating new insights and opportunities within academia and the start-up/SME communities and in so doing also expanding the expert workforce that will be required to facilitate future industrialisation. Funding systems such as administered by ‘DARPA’ in the US [68] provide such vital support, and are widely recognised within the start-up community to be invaluable in developing the foundations for future growth [69].

## Perspective

- By applying digital technologies with high throughput automated data generation combined within an engineering-inspired design-built-test-learn cycle process, synthetic biology is transforming the capability to redesign and engineer biological systems for industrial purposes. Complementing established methods and generating the potential to tackle increasingly challenging biological system designs, synthetic biology is poised to play an important role in assisting the translation of bioprocessing into industrial manufacture as sustainability challenges escalate.
- Notwithstanding the many applications successfully developed to date — predominantly in the healthcare sector — many technological challenges still need to be addressed via ongoing research to unlock the full potential for synthetic biology to contribute a pipeline of innovative solutions for IB. These include the ability to design increasingly complex cell functions leading to the development of robust engineered industrial hosts (‘chassis’) with predictable functionalities and the ability to predict industrial-scale operations more precisely from the laboratory and pilot-scale demonstrations. Unfamiliarity with the processes of synthetic biology within many established industries means that most applications are being developed within start-up companies and SMEs, the growth rates of which are highly dependent upon early-stage funding. There is a need to provide ‘long-tail’ public funding over many years (*cf*. the DARPA program) to ‘de-risk’ the field to the point where the private sector feels comfortable about investing substantially.
- Synthetic biology start-ups and SMEs are already starting to commercialise products and services within the IB sector, but to date, the bulk of funding has been into healthcare applications. The stage is set for many more applications to be commercialised, either via start-ups and SMEs growing independently or by established companies acquiring or developing relevant technologies in-house. This is being facilitated by the development of an expanding expert workforce familiar with the principles of responsible innovation and through the provision of access to an increasingly effective range of support facilities. Increasing consumer awareness of global sustainability challenges combined with government policies will continue to drive the need to develop innovative bio-based solutions for the foreseeable future. Synthetic biology is likely to play an ever-increasing role in future as a platform technology facilitating the delivery of the bioeconomy.

## Abbreviations

BSI: British Standards Institution
DBTL: design-build-test-learn
EBRC: engineering biology research consortium
HGP: Human Genome Project
IB: industrial biotechnology
RRI: responsible research and innovation
SBRCs: synthetic biology research centre

## Open Access

Open access for this article was enabled by the participation of Imperial College London in an all-inclusive *Read & Publish* pilot with Portland Press and the Biochemical Society under a transformative agreement with JISC.
